# Establishment and characterization of breast cancer organoids from a patient with mammary Paget’s disease

**DOI:** 10.1186/s12935-020-01459-6

**Published:** 2020-08-03

**Authors:** Bo Pan, Dongyi Zhao, Yaqian Liu, Na Li, Chen Song, Ning Li, Xuelu Li, Man Li, Zuowei Zhao

**Affiliations:** 1 Department of Oncology & Department of Breast Surgery, The Second Hospital of Dalian Medical University, Dalian, 116023 China; 2 Department of Foreign Language, Dalian Medical University, Dalian, 116000 China

**Keywords:** Breast cancer, Paget’s disease, Organoid culture, Genome sequencing

## Abstract

**Background:**

Mammary Paget’s disease (MPD) is an uncommon cutaneous intraepithelial malignancy with ulceration of the nipple or areola. Its pathogenesis and genomic mutation remain largely unknown and no cell lines are established from primary tumors.

**Methods:**

We collected surgical tumor specimens from a 65-year-old Chinese woman diagnosed with MPD and established patient-derived breast cancer (BC) organoids from MPD using organoid culture technology.

**Results:**

We successfully propagated BC organoids from a patient with MPD for more than 6 months. The organoids were cultured for long-term expansion without any change in spherical organoid morphology. Besides, the spherical organoid morphology did not change when they underwent cryopreservation after resuscitation. The H&E staining and immunohistochemistry analyses showed the similar morphological and histological features of the organoids compared with their paired original BC tissues. The organoids retained positive expression of breast cancer biomarkers: estrogen receptor, progesterone receptor, antigen Ki-67 and negative expression of human epidermal growth factor receptor 2. We also showed that MPD organoids recapitulated the unique genomic landscape including copy number alterations, mutational load, mutational signatures and cancer gene mutations by whole genome sequencing. In situ senescence-associated acid beta galactosidase assay confirmed senescence phenomenon existed in the process of organoids culture and there was no significant difference in the proportion of senescent organoids after organoid passage and resuscitation.

**Conclusions:**

Our results suggested that an effective platform for ex vivo BC organoids from MPD patients could be used to explore clinicopathological and genomic characteristics of these patients.

## Background

Paget’s disease (PD) is an uncommon cutaneous intraepithelial malignancy characterized histopathologically by large epidermal adenocarcinoma cells (Paget’s cells) containing abundant mucin [1]. According to the affected anatomic locations, PD is classified as mammary Paget’s disease (MPD) and extramammary Paget’s disease (EMPD). MPD is characterized by the eczematous eruption and ulceration of the nipple or areola [2]. The MPD incidence of all breast cancer (BC) is approximately 1%, and more than 90% of Paget’s disease of the nipple is associated with underlying intraductal carcinoma (DCIS) or infiltrating duct carcinoma (IDC) [1, 3]. Recent studies have confirmed that MPD conjunction with invasive cancer had worse prognosis [4]. Based on the overall survival, MPD patients with PD-IDC had worse prognosis (5-year survival rate = 84.1%) compared with MPD patients with PD-DCIS (5-year survival rate = 97.5%) [5]. The pathogenesis of PD has implications for the optimal treatment. The exact pathogenesis of the carcinoma of the epidermis is poorly understood, and debated as to whether it arises from the underlying breast parenchyma or the epidermal cells. There are two main theories proposed including the epidermotropic theory and the intraepidermal origin theory [6]. Epidermotropic theory declares that Paget’s cells originate from underlying carcinoma cells that migrate into the epidermis, whereas the intraepidermal origin theory states that Paget’s cells are the result of in situ oncogenic changes in epidermal cells of the apocrine gland ducts or pluripotent keratinocyte stem cells [6].

Recently, Zhang et al. [7] performed whole-exome sequencing on 41 pairs of PD tumor and normal skin samples to reveal the molecular landscapes of PD. They found that MPD and EMPD had the similar genomic aberrations, especially in genes involved in chromatin remodeling processes, such as *KMT2C* and *ARID2*. Further, MPD and underlying breast ductal carcinomas are likely independent oncogenic events. Gatalica et al. [8] investigated the molecular differences between MPD and EMPD, and found that *PIK3CA* and *TP53* mutations were most common in MPD. Although rare, several other genomic alterations were also detected in MPD. However, the full genomic mutational landscapes of MPD remain uncharacterized and genomics-related research is still scarce. For less frequently occurring BC or special pathological types, due to the lack of a corresponding pre-clinical cell culture model, it is difficult to study the tumorigenesis, the phenotypic and genetic heterogeneity of this type of BC which hampers therapeutic innovation. We need to develop a viable and reliable method to improve the therapeutic effects of BC patients with PD.

Three-dimensional (3D) organoid culture models open opportunities for both fundamental and translational cancer research. The organoids can be grown from primary patient material of a wide range of tumor tissues, such as kidney [9], colorectal [10], pancreas [11], lung [12] and breast cancer [13]. Tumor-derived organoids recapitulate and maintain the genetic heterogeneity of native tumor tissue over time, and have predictive value for individual patient drug responses [9, 10]. We previously performed two-dimensional (2D) culture technology to culture tumor cells from endometrial cancer patients [14] and breast cancer patients with leptomeningeal metastasis [15]. However, the tumor cells gradually underwent senescence after six or seven passages and could not expand as long as cancer cell lines. Afterwards, we successfully established a BC tissue-derived organoid of papillary carcinoma which had been continuously propagated for more than 6 months by using the organoid culture method [16]. It is important for us to study molecular pathogenesis and pathophysiology of uncommon pathological types BC as above. In this study, we describe the case of a 65-year-old Chinese woman with MPD and attempt to culture MPD-derived tumor cells using organoid culture method. To the best of our knowledge, this is the first report that presents the establishment of MPD patient-derived organoids.

## Materials and method

### Patient and sample collection

**Fig. 1:**
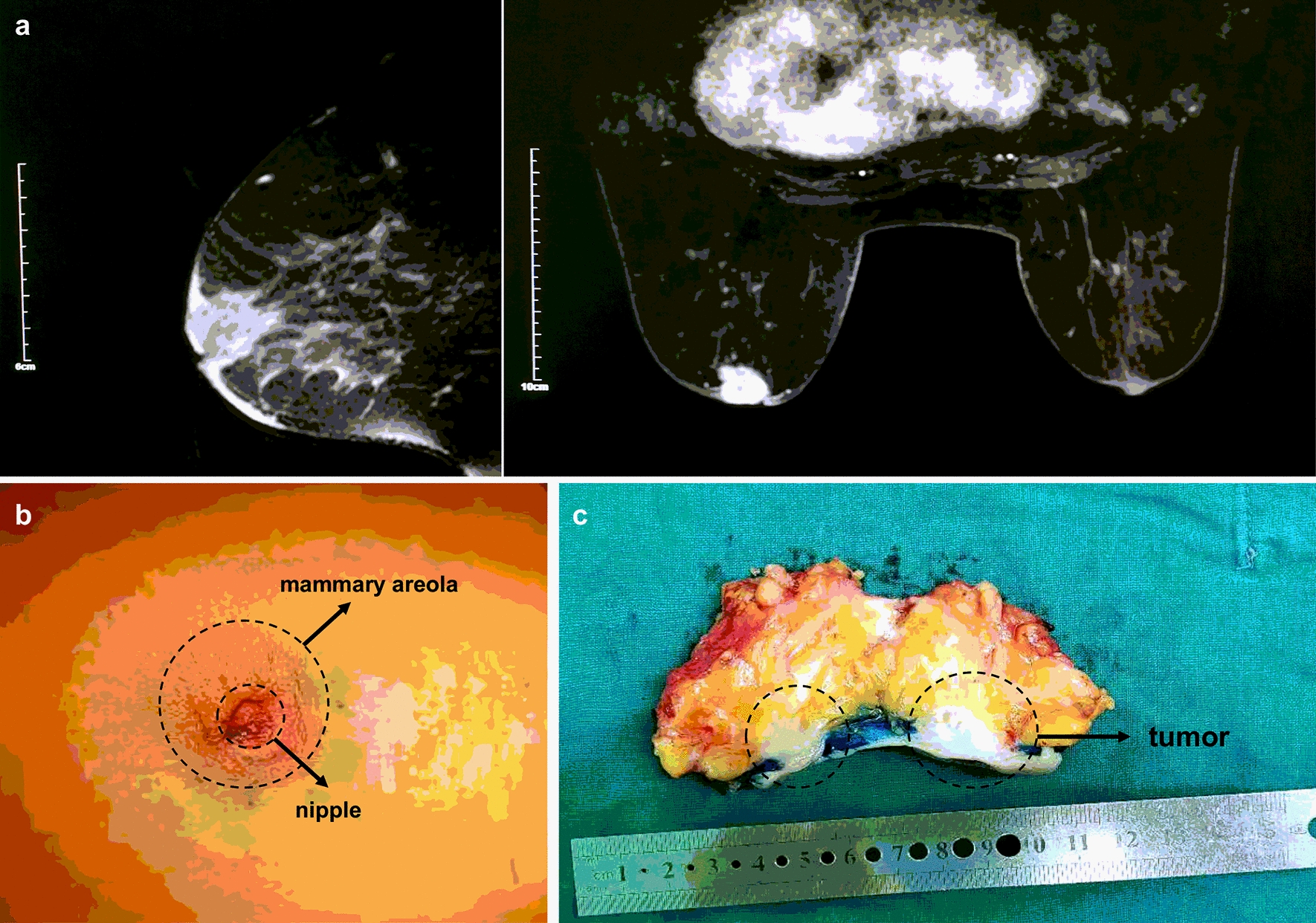
MRI scan, visual examination and surgical specimen of mammary Paget’s disease of right breast in a 65-year-old Chinese woman. **a** Chest MRI showed nodules in the left areola area with sunken skin. **b** Rupture of the left nipple was 0.3 × 0.3 cm with local blood exudation. **c** The gross pathologic inspection showed mammary glands and attached skin. MRI: Magnetic resonance imaging

A 65-year-old Chinese woman was admitted with nipple change and mass in her left breast. The patient complained that molting of left nipple with small amount of white exudation for 7 months and rupture of left nipple 1 day ago. Broad bean sized mass was found in her left breast near the nipple 5 months ago. The patient had no family history. Physical examination revealed a hard mass under the nipple and areola, with unclear borders and irregular shapes. Rupture of the left nipple was 0.3 × 0.3 cm with local blood exudation (Fig. 1b). The contralateral breast was normal and no axillary lymph nodes were palpable. The magnetic resonance imaging demonstrated that the mass in the left areola area with sunken skin, BI-RADS level 5 (Fig. 1a). The other examinations were negative and no distant metastases. The patient underwent modified radical mastectomy.

**Fig. 2:**
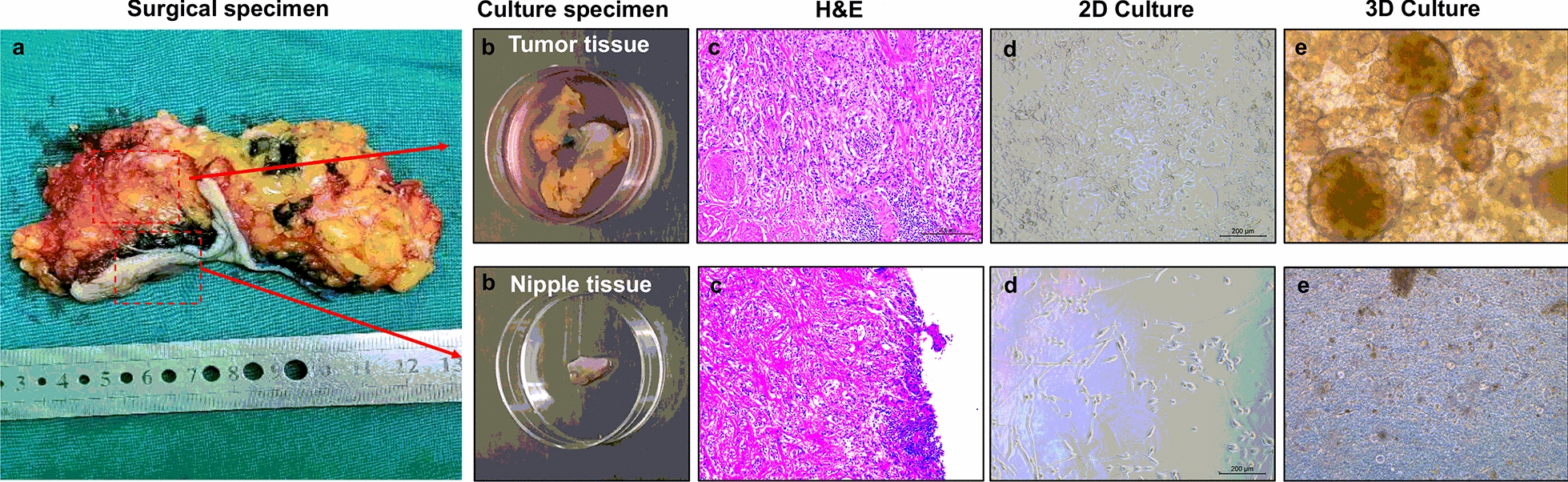
Representative images of organoids cultured from a mammary Paget’s disease patient. **a** Surgical specimen of mammary Paget’s disease. **b** Cut an appropriate size of nipple tumor tissue and breast tumor tissue behind the nipple for culture and store respectively. **c** Postoperative pathology results reported that both the nipple area and the mass behind the nipple were adenocarcinoma. **d**, **e** Culture tumor cells and organoids using nipple tumor tissue and breast tumor tissue under 2D and 3D cultivating mode separately

The postoperative gross pathologic inspection showed mammary glands and attached skin. Nipple was inverted, gray and tough swelling could be seen under the skin (Fig. 1c). Postoperative pathology results reported that both the nipple area and the mass behind the nipple were adenocarcinoma (Figs. 2 and 4). No axillary lymph node metastasis (0/24). The immunohistochemistry pathology further confirmed the high expression of estrogen receptor (ER) and progesterone receptor (PR) (Fig. 4). This patient is currently receiving aromatase inhibitor Letrozole. At 1-year follow-up, the patient was free of disease.

The tumor sample was obtained from the MPD patient at the time of surgery. The BC tissue was treated as we had described before [16]. The study was conducted at The Second Hospital of Dalian Medical University (Dalian, China). The research protocol was approved and recorded by the Ethics Committee of The Second Hospital of Dalian Medical University. All procedures are carried out in accordance with the Helsinki Declaration.

### Organoids and tumor cells culture

The tumor tissue was treated as we had described before [16]. The nipple tissue and tumor tissue was cut into pieces separately, washed with AdDF+++ (Advanced DMEM/F12 (Sigma, Saint Louis, MO, USA) containing 1× Glutamax (Invitrogen, Carlsbad, CA, USA), 10 mM HEPES (Invitrogen, Carlsbad, CA, USA) and 100 U/mL/100 mg/mL Penicillin/Streptomycin (Sigma, Saint Louis, MO, USA)) and digested in 3 mL medium [16] containing 1 mg/mL Collagenase (Sigma, Saint Louis, MO, USA) at 37 °C for appropriate time. The digested tissue was collected in 3 mL AdDF+++. If a red precipitate is formed, red blood cells are lysed in 1 mL of red blood cell lysis buffer (Roche, Basel, Switzerland) for 3 min at room temperature and centrifuged at 1300 rpm after adding 3 mL of AdDF +++.

3D organoid culture model: The pellet was suspended in cold Cultrex growth factor reduced BME type 2 (Trevigen, Gaithersburg, MD, USA). 45 μL of BME-cell suspension droplets were seeded in a preheated 24-well suspension plate (Corning Incorporated, NY, USA) at 37 °C for 25 min. After gelation was completed, it was overlaid with 450 μL medium. Passaging of organoids was performed as we had described before [16].

2D tumor cell culture model: The pellet was counted and 4 × 10^4^ of tumor cells were seeded on 24-well plates (Nest, Wuxi, Jiangsu, China) with 500 μL medium. The medium was changed and passaged every 3 days: 500 μL of TrypLE Express (Invitrogen, Carlsbad, CA, USA) was added to the tumor cells which incubated for 5 min at 37 °C. 1.5 mL AdDF +++ was added and centrifugated at 1000 rpm. The pellet was reseeded in the ratio (1:3) as described above.

### Immunohistochemistry

To maintain the 3D structure of the primary culture tumor cells, BME-cells mixture was aspirated from the 24-well plates gently and completely, then it was embedded in HistoGel (Biocoat, Corning, NY, USA). All samples were fixed in 4% paraformaldehyde before embedded in paraffin. The slides were incubated overnight with primary antibody Rabbit anti-ER antibody (1:5, ab27595), anti-PR (1:150, ab63605), anti-HER2 (1:600, ab134182), and anti-Ki67 (1:150, ab16667) which were purchased from Abcam (Cambridge, MA, UK). The DAB kit was purchased from Zhongshan Goldenbridge Biotechnology Company (Beijing, China). All procedures were carried out according to the manufacturer’s instructions.

### Genomic DNA analysis

The organoids and the oral epithelial cells were used for extraction of genomic DNA respectively, and subjected to whole genome sequencing by sequencing company (Shihe Gene, Nanjing, China). The detailed methods are described in Additional file 1: Data S1.

### In situ senescence-associated acid beta galactosidase assay

Cell senescence β-galactosidase staining kit was purchased from Beyotime (Shanghai, China). Organoids grown in 24-well suspension plate (Corning Incorporated, NY, USA) were fixed using β-galactosidase staining fixative for 15 min at room temperature. After that, the organoids were incubated with a dyeing working fluid at 37 °C overnight. All procedures were carried out according to the manufacturer’s instructions. The percentage of senescent cells was calculated by the number of blue, β-galactosidase-positive cells out of at least 500 cells in different microscope fields, as already reported [17].

## Results

### Establishing MPD patient-derived organoids

**Fig. 3:**
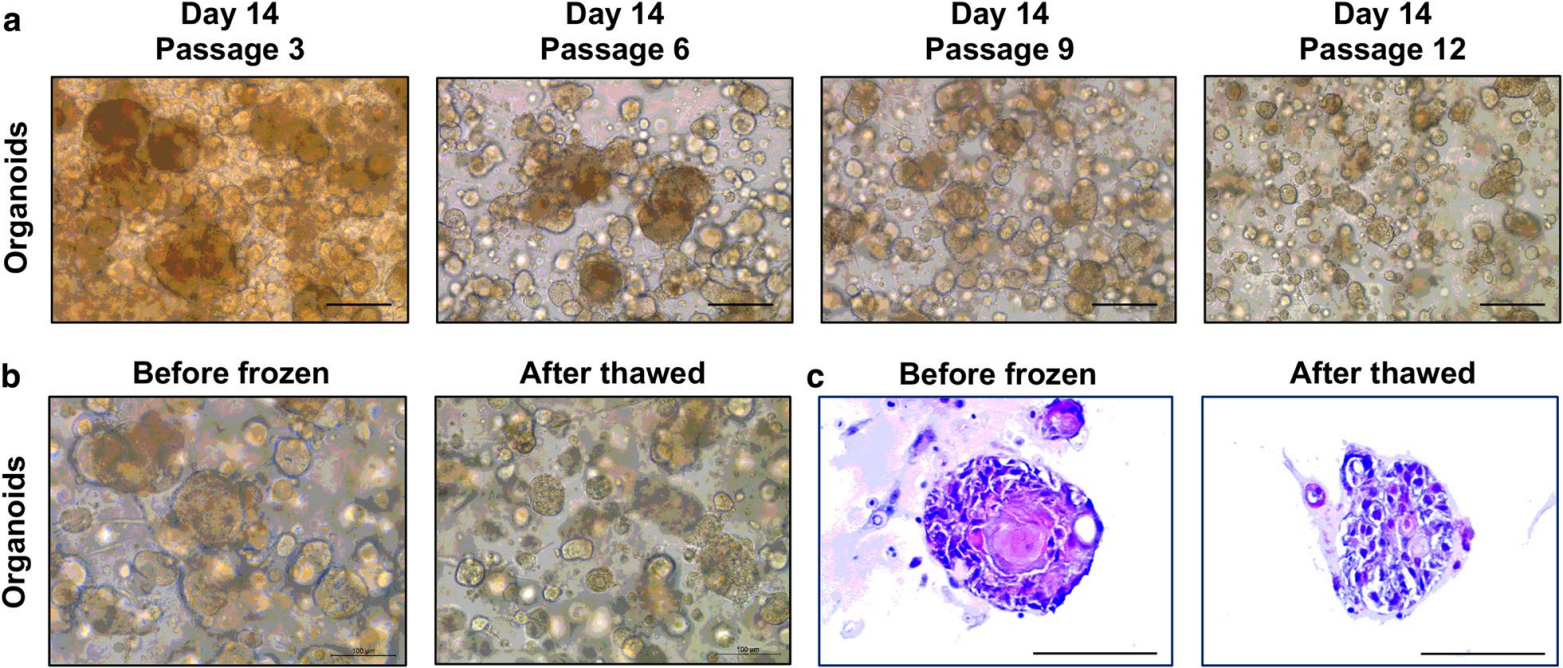
Breast cancer organoids established from a mammary Paget’s disease patient. **a** Representative images of long-term cultured breast cancer organoids without change in spherical organoid morphology. **b**, **c** The morphology of breast cancer organoids did not change before freezing and after thawing by Bright-field microscopy images and H&E staining. Scale bar = 100 μm

In order to evaluate the feasibility of primary culture of MPD-derived tumor cells, we used organoid technology to culture these tumor cells from our patient. Due to the consistency of the time and space of the nipple tumor and the tumor behind the nipple areola, we thought their tumor cells were likely to have the same origin. We obtained BC surgical specimen from this patient who underwent the mastectomy (Fig. 1b and c). We used nipple tumor tissue and tumor tissue behind the nipple to culture tumor cells under 2D cultivating mode and organoids under 3D cultivating mode separately. However, we failed to culture nipple tissue-derived tumor cells under both 2D and 3D condition. We only observed fibroblasts and it was difficult to observe tumor cells under 2D condition (Fig. 2). The culture state of nipple tissue-derived tumor cells under 3D condition was poor in the process of culture and died gradually even after passage (Fig. 2). On the other hand, we successfully cultured the tumor tissue behind the nipple-derived tumor cells under both 2D and 3D condition (Figs. 2 and 3). The tumor cells grew rapidly under 2D condition and the cultivation process at 1 day, 4 days and 7 days was recorded. When they were cultured for 4 days, they began to grow fast and fill up the entire cultivation space quickly after 4 days of cultivation (Additional file 2: Figure S1). The organoids grew relatively slowly under 3D condition and we recorded the cultivation process of day 1, 5, 10 and 15. When they were cultured under 3D condition for 10 days, they began to fill up the entire cultivation space quickly. The number of organoid masses increased rapidly and the volume increased gradually (Additional file 2: Figure S1). We observed dense and solid organoids and they were cohesive. (Figs. 2 and 3). After the organoids were cultured for 15 days, it could be seen that the growth of the organoids was obviously slow down and there was no significant change in the number and size of organoids (Additional file 2: Figure S1). The organoids were cultured for long-term expansion without any change in spherical organoid morphology (Fig. 3). Besides, the spherical organoid morphology did not change when they underwent cryopreservation after resuscitation (Fig. 3). We successfully established the MPD patient-derived organoids which had been continuously propagated for more than 6 months.

### MPD patient-derived organoids match the original histological characteristics

**Fig. 4:**
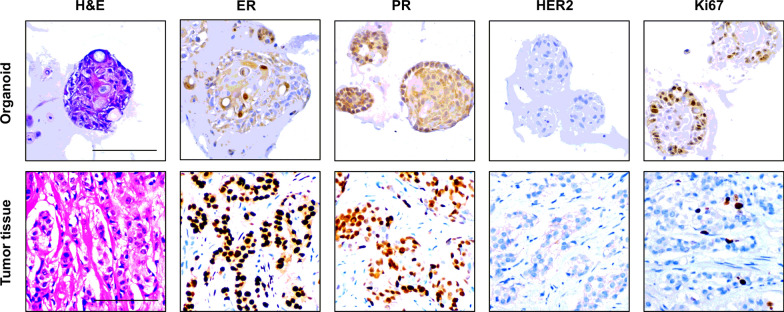
Histology and receptor status of breast cancer organoids. Comparative histological and immunohistochemical images of breast cancer organoids and their original breast cancer tissues. Histology and breast cancer biomarkers (ER, PR, HER2, Ki67) were similarly well retained in the organoids

We performed histopathological analysis of H&E stained tissues and MPD patient-derived organoids sections, and confirmed that the phenotypes of tumor cells matched the histological characteristics of BC. Based on cellular and nuclear atypia, tumor cells clearly showed malignant characteristics. The cell size was also different and enlarged (Fig. 4). The spherical organoid morphology did not change when they underwent cryopreservation after resuscitation by H&E staining (Fig. 3). Besides histological conservation, the organoids retained ER and PR positive expression of the BC biomarkers like primary BC and negative expression of human epidermal growth factor receptor 2 (HER2) (Fig. 4). The analysis of Immunohistochemistry also showed strongly positive staining of antigen Ki-67 (Fig. 4). Together, we found that MPD patient-derived organoids matched the primary tumor in histopathology and hormone receptor status, and could be a high-fidelity model.

### Genomic characterization of MPD patient-derived organoids

**Fig. 5:**
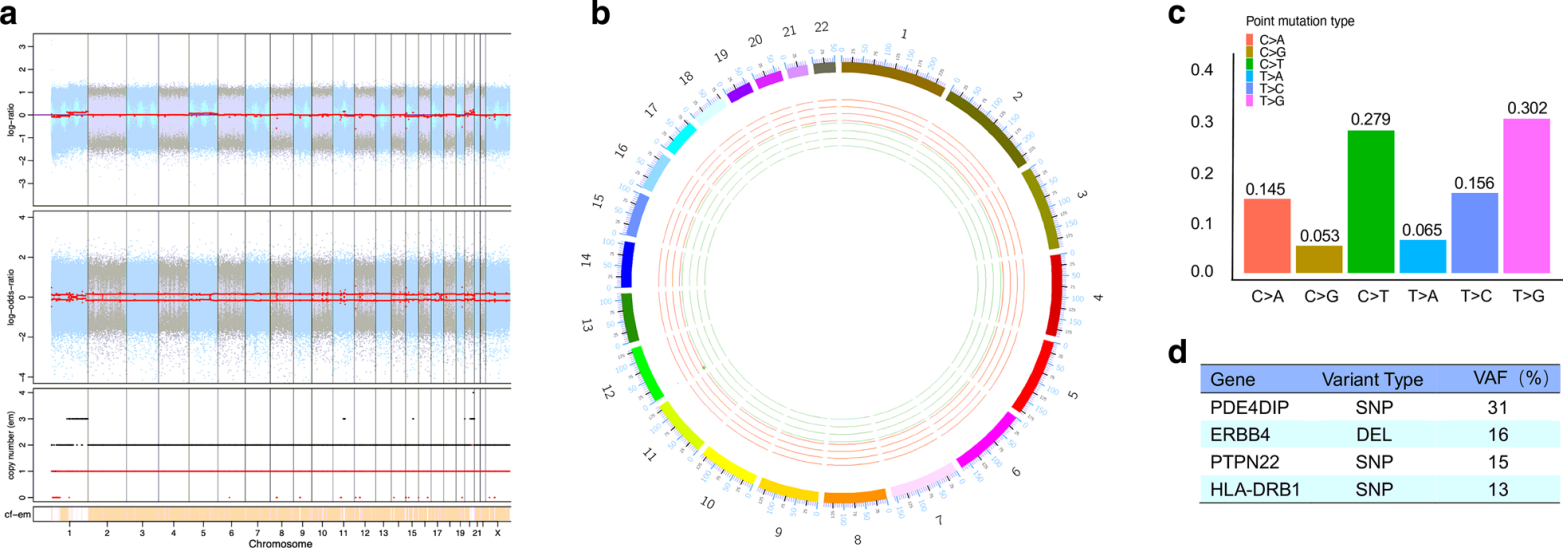
Genomic Characterization of the organoids derived from a mammary Paget’s disease patient. **a**, **b** Scatterplot and circos plot illustrating genome copy number alterations of the organoids which showed clean copy number signals and changes in copy number gains or losses were not obvious. **c** Bar graph showing the relative contributions of point mutation types underlying mutational signatures for the organoids. **d** Somatic mutations found in breast cancer genes of the organoids

Whole genome sequencing of oral epithelial cells and MPD patient-derived organoids was used to characterize genomes of MPD patient-derived organoids including copy number alterations, mutational signatures and somatic mutations. The organoids showed clean copy number signals and changes in copy number gains or losses were not obvious (Fig. 5a, b and Additional file 3: Figure S2). We analyzed base substitutions in organoids and plotted the total number of mutations per mutational signature. We found the organoids with more than 250 mutations in total as well as the relative contribution of individual signatures (Additional file 3: Figure S2). We identified significant contributions of major BC signatures in the organoids (Additional file 3: Figure S2), including Signature 1, Signature 3 and Signature 30. The point mutation type was dominated by T > G and C > T (Fig. 5c). Besides, the somatic mutations of the organoids were identified (Additional file 4: Data S2) and cancer mutant genes in the most relevant BC genes were also found, including *ERBB4*, *HLA*-*DRB1*, *PDE4DIP*, *PTPN22* (Fig. 5d).

### Senescence is present in MPD patient-derived organoids

To evaluate the presence of cellular senescence in breast cancer cells, we used MPD patient-derived organoids to perform in situ senescence-associated acid beta galactosidase assay. We selected the 3rd generation organoids, the 12th generation organoids and the resuscitated organoids for evaluation of the effect of passage and resuscitation on organoid senescence. Senescence organoid clusters were found in all three organoid cultures and larger organoid clumps were more stained. However, there were no significant statistical differences among them (Additional file 2: Figure S1). Therefore, senescence phenomenon existed in the process of organoids culture and there was no significant difference in the proportion of senescent organoids after organoid passage and resuscitation.

## Discussion

Here we describe an effective platform for ex vivo expansion of BC tumor cells derived from a MPD patient. To our knowledge, this is the first report that presents the establishment of MPD patient-derived organoids. We proved the consistency of the morphological and histological features between the organoids and its original BC tissues by performing H&E staining and immunohistochemistry analysis. We found novel genomic characteristics of MPD patient-derived organoids including copy number alterations, mutational signatures and somatic mutations by whole genome sequencing. This platform could be used to explore clinicopathological and genomic characteristics of these patients.

At present, the precise origin of mammary Paget’s cells has been debated for a long time. It has been proposed that they arise within the epidermis (intraepidermal theory) or from underlying breast carcinomas (epidermotropic theory) [6]. In this study, we tried to use nipple tumor tissue and tumor tissue behind the nipple to culture organoids. We failed to culture nipple tumor tissue-derived tumor cells, and too few nipple-derived tumor cells were the cause of cultivation failure. At present, no previous studies had reported primary culture of nipple tissue-derived tumor cells successfully and the proper culture conditions were not known. However, we successfully established BC organoids from tumor tissue near the nipple which had been continuously propagated for more than 6 months. We found that its unique morphological characteristics and the bright-field morphology of MPD patient-derived organoids were different from that of previously reported breast cancer patients [13, 16]. We observed dense and solid organoids and they were cohesive. The organoids were cultured for long-term expansion without any change in spherical organoid morphology. The spherical organoid morphology did not change when they underwent cryopreservation after resuscitation. We thought this was a breakthrough in the field of primary cell culture of MPD.

Recent studies [18] found that the molecular subtype of the associated BC is usually similar to that of MPD. The HER2-enriched subtype is the most frequently occurring molecular subtype in MPD, followed by the luminal subtype. In this study, we successfully established BC organoids from a Luminal A subtype BC patient with MPD. We performed histopathological analysis of H&E stained tissues and MPD-derived organoids sections, and confirmed that the organoids matched the histological characteristics of MPD by H&E staining. Besides histological conservation, the organoids retained ER and PR positive expression of the breast cancer biomarkers like primary BC. The analysis of immunohistochemistry also showed strongly positive staining of Ki67.

The full genomic mutational landscapes of MPD remain uncharacterized, and genomics-related research is still scarce. Zhang et al. [7] performed whole-exome sequencing on 41 pairs of PD tumor and normal skin samples to reveal the molecular landscapes of PD. They found that MPD and EMPD were shown to have similar genomic aberrations, especially in genes involved in chromatin remodeling processes, such as *KMT2C* and *ARID2*. Further, MPD and underlying breast ductal carcinomas are likely independent oncogenic events. *KMT2C* is likely an early oncogenic driver for PD. Gatalica et al. [8] investigated the molecular differences between MPD and EMPD. They found *PIK3CA* and *TP53* mutations were most common in MPD. Although rare, several other genomic alterations were also detected in MPD, including *CHEK2*, *CDK12*, *MLLT6* and *MDM2*. Copy number alterations affect a larger fraction of the cancer genome than any other type of genetic alterations [6]. Recent research found BC organoids recapitulated the original copy number alterations patterns of cancer genes and showed signal amplitude similarly to genome-wide copy number alterations specific BC genes. Copy number alterations were retained across several tumor-organoid pairs include CDKN2A, ERBB2, NF1, and SNX31 [13]. We employed whole genome sequencing to characterize genomes of MPD-derived organoids including copy number alterations, mutational signatures and somatic mutations. We found that the organoids showed clean copy number signals and changes in copy number gains or losses were not obvious.

We analyzed base substitutions in organoids and plotted the total number of mutations per mutational signature. We found the organoids with more than 250 mutations in total as well as the relative contribution of individual signatures. Diverse mutational processes result in distinct mutational signatures, at least 12 of which are found in BC [19, 20]. We identified significant contributions of major BC signatures in the organoids, including Signature 1, Signature 3 and Signature 30. Signature 1 [19, 20] is the result of an endogenous mutational process initiated by spontaneous deamination of 5-methylcytosine. It is associated with small numbers of small insertions and deletions in most tissue types which correlates with age of cancer diagnosis. Signature 3 [19, 20] is associated with failure of DNA double-strand break-repair by homologous recombination. It has relationship with elevated numbers of large (longer than 3 bp) insertions and deletions with overlapping microhomology at breakpoint junctions. It also associated with germline and somatic *BRCA1* and *BRCA2* mutations in breast. Signature 30 [19, 20] has been observed in a small subset of breast cancers and the aetiology of Signature 30 remains unknown.

The point mutation type was dominated by T > G and C > T and similar to previously described mutational signatures for PD [7]. The somatic mutations of the organoids were identified and cancer genes in the most relevant BC genes were also found, including *ERBB4*, *HLA*-*DRB1*, *PDE4DIP*, *PTPN22*.

It is similar to the previously described research that BC organoids displayed mutations in many of the most relevant BC genes [13].

Generally, senescent cells are observed as human beings age. They are also observed in normal organ development and pathological conditions related to aging [21, 22]. Most of the research on senescent cells has been conducted in primary isolated normal cells, and in vitro studies have induced cellular senescence mainly through DNA stimulation or subculture of fibroblasts [23]. Recently, cellular senescence has been observed in tumor tissues and may be involved in cancer progression [24, 25]. These senescent cells are thought to originate from tumor cells and are called senescent tumor cells [26]. Senescent tumor cells are observed in various carcinomas [27, 28] but it was rarely reported in breast cancer. In some carcinomas, the distribution of senescent tumor cells is not even, and it is observed in specific tumor tissue locations. Senescent tumor cells do not exist in the center of the mass where hypoxic damage usually occurs, but rather in the marginal region of the tumor [28]. Furthermore, they are present in large numbers in the metastatic lymph nodes and lymphatic vessels [28]. These data demonstrate that senescent tumor cells are involved in cancer progression. To evaluate the presence of cellular senescence in primary cultured breast cancer cells, we used MPD patient-derived organoids to perform in situ senescence-associated acid beta galactosidase assay. We selected the 3rd generation organoids, the 12th generation organoids and the resuscitated organoids for evaluation of the effect of passage and resuscitation on organoid senescence. Senescence organoid clusters were found in all three organoid cultures and larger organoid clumps were more stained. However, there were no significant statistical differences among them. Therefore, senescence phenomenon existed in the process of organoids culture and there was no significant difference in the proportion of senescent organoids after organoid passage and resuscitation. The major limitation of the current study is due to the analysis of only one patient. The effect of senescent tumor cells on tumors and tumour progression remains controversial which is still to be further studied. In the future, we plan to build a larger-scale organoid model of MPD patients which will contribute to elucidation of pathophysiological mechanisms of MPD. It accelerates both basic and translational research and promotes better prognosis for patients with MPE.

## Conclusion

We successfully established a novel BC organoid model from a MPD patient which could be used to explore clinicopathological and genomic characteristics of these patients.

## Supplementary information

**Additional file 1: Data S1.** The experimental procedure of whole genome sequencing.

**Additional file 2: Figure S1.** Representative images of successful 2D and 3D growth process and senescence evaluation of the organoids derived from a mammary Paget’s disease patient. a. The tumor cells grew rapidly under 2D condition and the cultivation process at 1 day, 4 days and 7 days was recorded. They began to fill up the entire cultivation space quickly after 4 days of cultivation. Scale bar = 200 μm. b. The organoids grew relatively slowly under 3D condition and we recorded the cultivation process of day 1, 5, 10 and 15. When they were cultured under 3D condition for 10 days, they began to fill up the entire cultivation space quickly. Scale bar = 200 μm. c. We found the phenomenon of senescence during organoid culture. Senescence phenomenon existed in the process of organoids culture and there was no significant difference in the proportion of senescent organoids after organoid passage and resuscitation. Scale bar = 100 μm. The graph shows the mean percentage of the senescent 3rd generation organoids, the 12th generation organoids and the resuscitated organoids. Mean ± SD of results from 3 independent field of microscope is shown.

**Additional file 3: Figure S2.** Genomic Characterization of the organoids derived from a mammary Paget’s disease patient. a. Heatmap showing copy number alterations in coding DNA sequences of breast cancer genes. b. Stacked bar graph showing the total mutation load per mutational signature of the organoids. Typical breast cancer mutational signatures (bold) were present and conserved.

**Additional file 4: Data S2.** The somatic mutations of the organoids.

## Data Availability

All data during this research are included in this published article.

## Abbreviations

PD: Paget’s disease
MPD: Mammary Paget’s disease
EMPD: Extramammary Paget’s disease
BC: Breast cancer
DCIS: Intraductal carcinoma
IDC: Infiltrating duct carcinoma
3D: Three-dimensional
2D: Two-dimensional
ER: Estrogen receptor
PR: Progesterone receptor
HER2: Human epidermal growth factor receptor 2
Ki67: Antigen Ki-67
