# Supplementary material for: Establishment and characterization of breast cancer organoids from a patient with mammary Paget’s disease
Source: Cancer Cell Int. 2020 Aug 3;20:365. doi: 10.1186/s12935-020-01459-6 (PMC7397673; doi:10.1186/s12935-020-01459-6)
Supplement: Supplementary file 1 — Additional file 1: Data S1. The experimental procedure of whole genome sequencing. [file 12935_2020_1459_MOESM1_ESM.docx]

**SUPPORTING INFORMATION**

**Genomic DNA analysis**

The quality of isolated genomic DNA was verified by using these two methods in combination: (1) DNA degradation and contamination were monitored on 1% agarose gels. (2) DNA concentration was measured by Qubit® DNA Assay Kit in Qubit® 2.0 Flurometer (Invitrogen, USA). A total amount of 0.5 μg DNA per sample was used as input material for the DNA library preparations. Sequencing library was generated using Truseq Nano DNA HT Sample Prep Kit (Illumina USA) following manufacturer’s recommendations and index codes were added to each sample. Briefly, genomic DNA sample was fragmented by sonication to a size of 350 bp. Then DNA fragments were endpolished, A-tailed, and ligated with the full-length adapter for Illumina sequencing, followed by further PCR amplification. After PCR products were purified (AMPure XP system), libraries were analyzed for size distribution by Agilent 2100 Bioanalyzer and quantified by real-time PCR (3nM). The clustering of the index-coded samples was performed on a cBot Cluster Generation System using Hiseq X PE Cluster Kit V2.5 (Illumina) according to the manufacturer’s instructions. After cluster generation, the DNA libraries were sequenced on Illumina Hiseq platform and 150 bp paired-end reads were generated. The steps of quality control processing were as follows: (1) Discard a paired reads if either one read contains adapter contamination (>10 nucleotides aligned to the adapter, allowing ≤ 10% mismatches); (2) Discard a paired reads if more than 10% of bases are uncertain in either one read; (3) Discard a paired reads if the proportion of low quality (Phred quality <5) bases is over 50% in either one read.

All the downstream bioinformatics analyses were based on the high quality clean data, which were retained after these steps. At the same time, QC statistics including total reads number, raw data, raw depth, sequencing error rate, percentage of reads with Q30 (the percent of bases with phred-scaled quality scores greater than 30) and GC content distribution were calculated and summarized. Valid sequencing data was mapped to the reference human genome (UCSC hg19) by Burrows-Wheeler Aligner (BWA) software [1] to get the original mapping results stored in BAM format. If one or one paired read(s) were mapped to multiple positions, the strategy adopted by BWA was to choose the most likely placement. If two or more most likely placements presented, BWA picked one randomly. Then, SAMtools [2] and Picard (http://broadinstitute.github.io/picard/) were used to sort BAM files and do duplicate marking, local realignment, and base quality recalibration to generate final BAM file for computation of the sequence coverage and depth. Mapping step was very difficult due to mismatches, including true mutation and sequencing error, and duplicates resulted from PCR amplification. These duplicate reads were uninformative and shouldn’t be considered as evidence for variants. We used Picard to mark these duplicates for follow up analysis. Samtools [2] mpileup and bcftools were used to do variant calling and identify SNP, InDels. Control-FREEC [3] was utilized to do CNV detection, while Crest [4] was specialized for SV discovery. Functional annotation was very important because the link between genetic variations and diseases would be clarified in this step. ANNOVAR [5] was performed to do annotation for VCF (Variant Call Format) obtained in the previous effort. dbSNP, 1000 Genome and other related existing databases were applied to characterize the detected variants. Given to the significance of exonic variants, gene transcript annotation databases, such as Consensus CDS, RefSeq, Ensembl and UCSC, were also included to determine amino acid alternation. Annotation content contained the variant position, variant type, conservative prediction, etc. These annotation results would help to locate disease causal mutant. The details of annotation were exhibited in supplemented material. The somatic SNV was detected by muTect [6], the somatic InDel by Strelka [7], and the somatic structural variants (SV) by CREST [8]. Control-FREEC was used to detect somatic CNV [9].

**Reference**

[1] Li H, Durbin R. Fast and accurate short read alignment with Burrows–Wheeler transform. Bioinformatics. 2009; 25: 1754-1760.

[2] Li H, Handsaker B, Wysoker A, et al. The sequence alignment/map format and SAMtools. Bioinformatics. 2009; 25: 2078-2079.

[3] Boeva V, Popova T, Bleakley K, et al. Control-FREEC: a tool for assessing copy number and allelic content using next-generation sequencing data. Bioinformatics. 2012; 28: 423-425.

[4] Wang J, Mullighan C G, Easton J, et al. CREST maps somatic structural variation in cancer genomes with base-pair resolution. Nature methods. 2011; 8: 652-654.

[5] Wang K, Li M, Hakonarson H. ANNOVAR: functional annotation of genetic variants from high-throughput sequencing data. Nucleic acids research. 2010; 38: e164.

[6] Cibulskis K, Lawrence MS, Carter SL, et al. Sensitive detection of somatic point mutations in impure and heterogeneous cancer samples. Nature biotechnology. 2013; 31: 213-219.

[7] Saunders CT, Wong WS, Swamy S, Becq J, Murray LJ, Cheetham RK: Strelka: accurate somatic small-variant calling from sequenced tumor-normal sample pairs. Bioinformatics. 2012; 28: 1811-1817.

[8] Wang J, Mullighan C G, Easton J, et al. CREST maps somatic structural variation in cancer genomes with base-pair resolution. Nature methods. 2011; 8: 652-654.

[9] Boeva V, Popova T, Bleakley K, et al. Control-FREEC: a tool for assessing copy number and allelic content using next-generation sequencing data. Bioinformatics. 2012; 28: 423-425.
