# Supplementary figures and images for: Establishment and characterization of breast cancer organoids from a patient with mammary Paget’s disease
Source: Cancer Cell Int. 2020 Aug 3;20:365. doi: 10.1186/s12935-020-01459-6 (PMC7397673; doi:10.1186/s12935-020-01459-6)

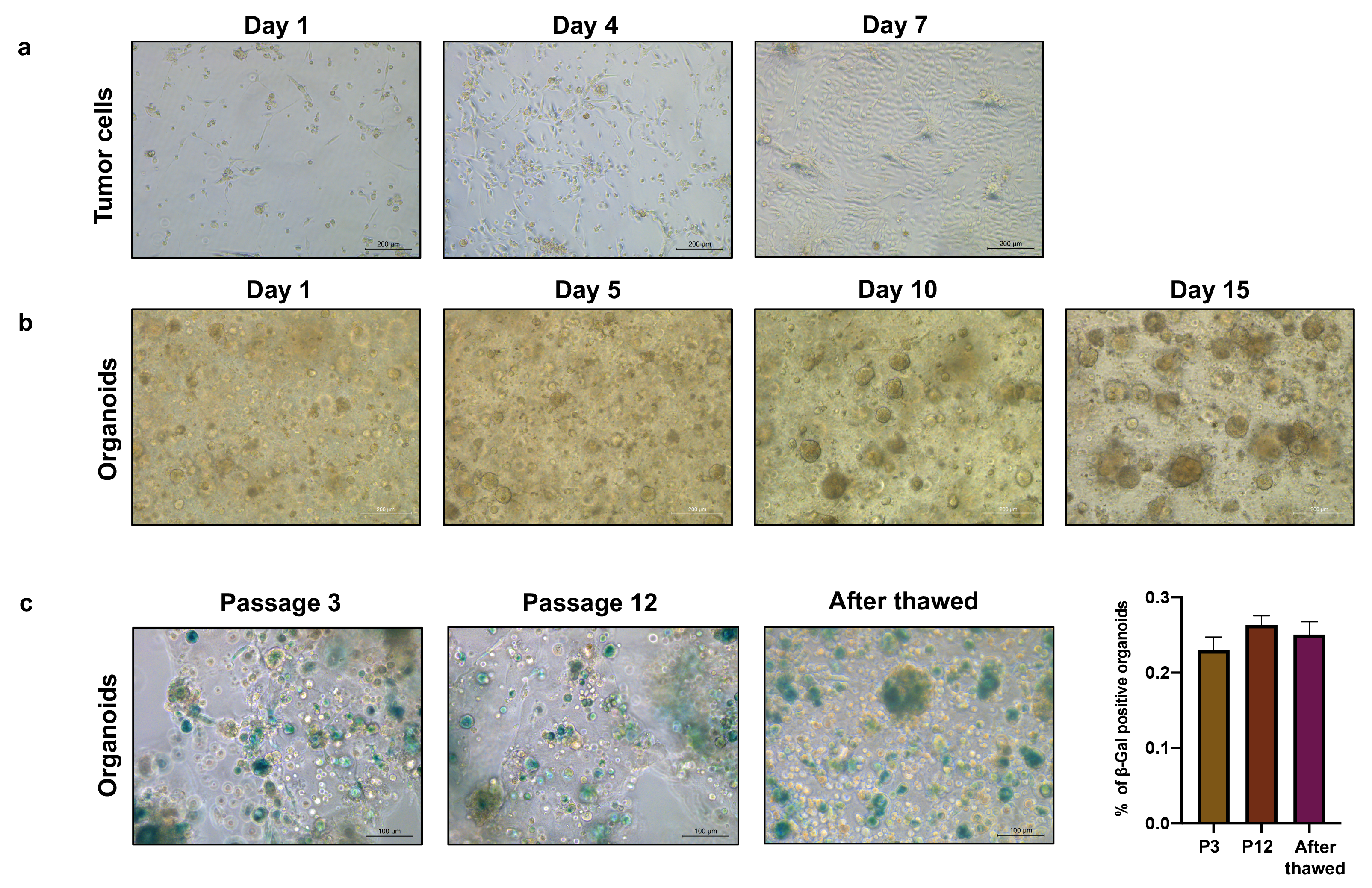

Supplement: Supplementary file 2 — Additional file 2: Figure S1. Representative images of successful 2D and 3D growth process and senescence evaluation of the organoids derived from a mammary Paget’s disease patient. a. The tumor cells grew rapidly under 2D condition and the cultivation process at 1 day, 4 days and 7 days was recorded. They began to fill up the entire cultivation space quickly after 4 days of cultivation. Scale bar = 200 μm. b. The organoids grew relatively slowly under 3D condition and we recorded the cultivation process of day 1, 5, 10 and 15. When they were cultured under 3D condition for 10 days, they began to fill up the entire cultivation space quickly. Scale bar = 200 μm. c. We found the phenomenon of senescence during organoid culture. Senescence phenomenon existed in the process of organoids culture and there was no significant difference in the proportion of senescent organoids after organoid passage and resuscitation. Scale bar = 100 μm. The graph shows the mean percentage of the senescent 3rd generation organoids, the 12th generation organoids and the resuscitated organoids. Mean ± SD of results from 3 independent field of microscope is shown. [file 12935_2020_1459_MOESM2_ESM.tiff]

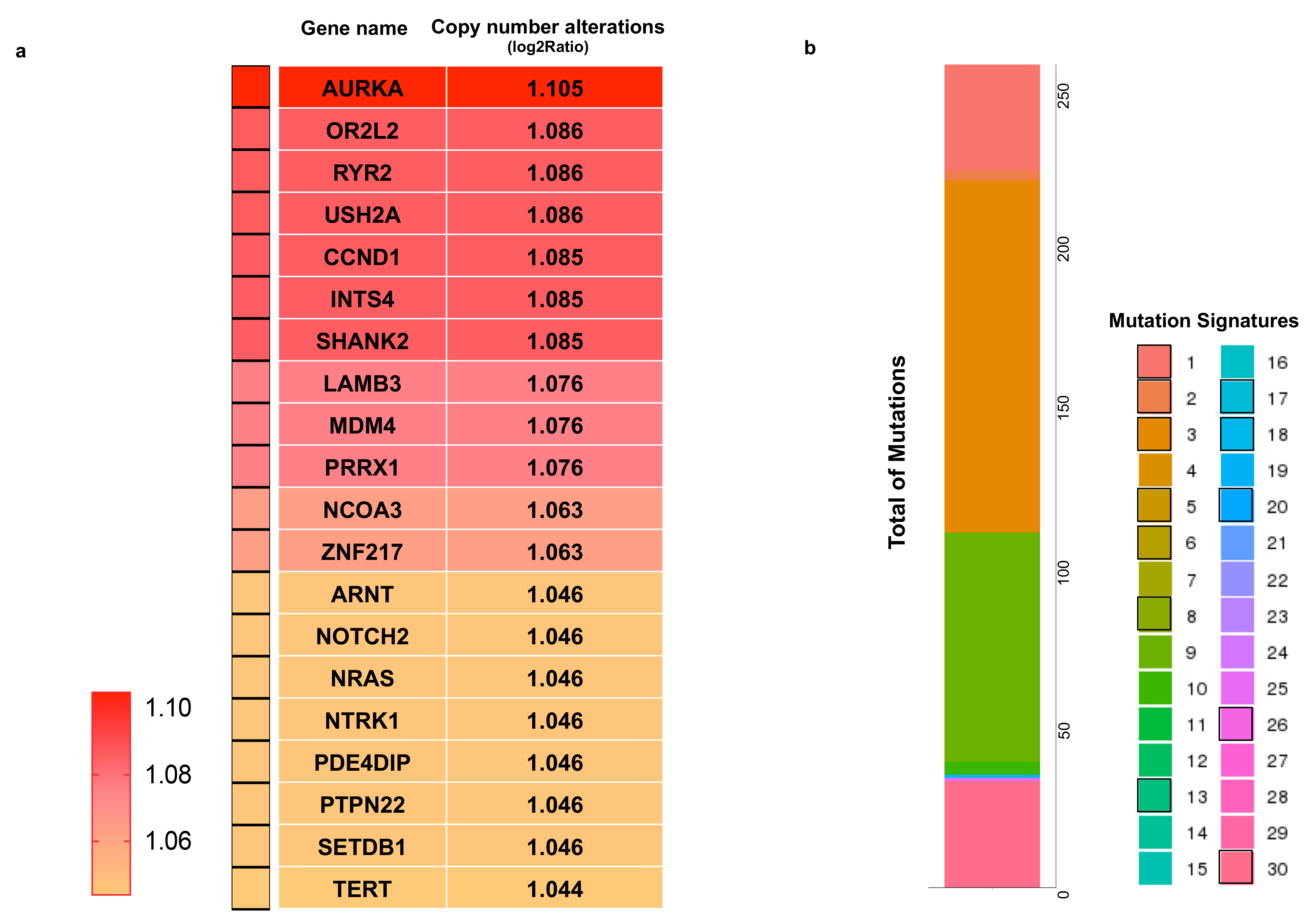

Supplement: Supplementary file 3 — Additional file 3: Figure S2. Genomic Characterization of the organoids derived from a mammary Paget’s disease patient. a. Heatmap showing copy number alterations in coding DNA sequences of breast cancer genes. b. Stacked bar graph showing the total mutation load per mutational signature of the organoids. Typical breast cancer mutational signatures (bold) were present and conserved. [file 12935_2020_1459_MOESM3_ESM.tiff]
